# The role of the N-terminal segment of CCR5 in HIV-1 Env-mediated membrane fusion and the mechanism of virus adaptation to CCR5 lacking this segment

**DOI:** 10.1186/1742-4690-4-55

**Published:** 2007-08-08

**Authors:** Gregory B Melikyan, Emily J Platt, David Kabat

**Affiliations:** 1 Institute of Human Virology, University of Maryland School of Medicine.725 W. Lombard St., Baltimore, MD 21201, USA; 2 Department of Biochemistry and Molecular Biology, Oregon Health and Science, University, Portland, OR 97239, USA

## Abstract

**Background:**

HIV-1 envelope glycoprotein (Env) induces membrane fusion as a result of sequential binding to CD4 and chemokine receptors (CCR5 or CXCR4). The critical determinants of CCR5 coreceptor function are the N-terminal domain (Nt) and the second extracellular loop. However, mutations in gp120 adapt HIV-1 to grow on cells expressing the N-terminally truncated CCR5(Δ18) (Platt *et al*., *J. Virol*. 2005, 79: 4357–68).

**Results:**

We have functionally characterized the adapted Env (designated Env(NYP)) using a quantitative cell-cell fusion assay. The rate of fusion with target cells expressing wild-type CCR5 and the resistance to fusion inhibitors was virtually identical for wild-type Env and Env(NYP), implying that the coreceptor affinity had not increased as a result of adaptation. In contrast, Env(NYP)-induced fusion with cells expressing CCR5(Δ18) occurred at a slower rate and was extremely sensitive to the CCR5 binding inhibitor, Sch-C. Resistance to Sch-C drastically increased after pre-incubation of Env(NYP)- and CCR5(Δ18)-expressing cells at a temperature that was not permissive to fusion. This indicates that ternary Env(NYP)-CD4-CCR5(Δ18) complexes accumulate at sub-threshold temperature and that low-affinity interactions with the truncated coreceptor are sufficient for triggering conformational changes in the gp41 of Env(NYP) but not in wild-type Env. We also demonstrated that the ability of CCR5(Δ18) to support fusion and infection mediated by wild-type Env can be partially reconstituted in the presence of a synthetic sulfated peptide corresponding to the CCR5 Nt. Pre-incubation of wild-type Env- and CCR5(Δ18)-expressing cells with the sulfated peptide at sub-threshold temperature markedly increased the efficiency of fusion.

**Conclusion:**

We propose that, upon binding the Nt region of CCR5, wild-type Env acquires the ability to productively engage the extracellular loop(s) of CCR5 – an event that triggers gp41 refolding and membrane merger. The adaptive mutations in Env(NYP) enable it to more readily release its hold on gp41, even when it interacts weakly with a severely damaged coreceptor in the absence of the sulfopeptide.

## Background

HIV-1 envelope glycoprotein (Env) initiates infection by promoting fusion between the viral and cellular membrane. Sequential binding of the gp120 subunit of Env to CD4 and a coreceptor (CCR5 or CXCR4) triggers conformational changes in the transmembrane subunit, gp41, which ultimately mediates membrane fusion [1,2]. The energy required to merge two membranes is, at least in part, released upon gp41 refolding from its native metastable conformation into the final six-helix bundle structure [3-5]. While HIV Env-induced membrane fusion has been extensively studied during the last two decades, the identity of gp41 conformational intermediates and the mode by which these intermediates are coupled to membrane rearrangements underlying the fusion process have not been fully elucidated.

One of the key steps of the fusion reaction is formation of an Env-CD4-coreceptor complex that initiates conformational changes in the spring-loaded [6] native structure of gp41. Engagement of coreceptors by Env is a complex, multistep process that involves several coreceptor domains [7-17]. Genetic analyses revealed that R5-tropic HIV-1 Env interact primarily with the N-terminal segment (Nt) and the second extracellular loop (ECL2) of CCR5 [11,18-22]. The gp120 domains that are involved in interactions with CCR5 are the V3-loop and the bridging sheet that is formed after gp120-CD4 binding and is comprised of conserved residues from the C4 domain and from the stems of variable V1/V2 and V3 loops [8,23-28]. The tip (often referred to as crown) of the V3-loop appears to interact with the ECL2, whereas the bridging sheet and the conserved residues of the V3 stem are likely to engage the Nt domain of CCR5 [8,16,23].

It has been shown that the acidic residues and sulfated Tyr within the Nt domain of CCR5 are important for HIV entry [18,29-31]. Thus proper post-translational modification of chemokine receptors is essential for their mediation of gp120 binding and infection. Interestingly, the CCR5 Nt-derived sulfated peptides are capable of reconstituting the function of the CCR5 mutant lacking the N-terminal segment [32]. Because these sulfated peptides specifically interact with R5-tropic Env in a CD4-dependent manner [32-34], the CCR5 Nt domain appears to be a critical determinant of the gp120 tropism. In contrast, X4-tropic isolates are generally less dependent on the Nt region of CXCR4 for their entry [32,35].

Even though the major domains involved in gp120-coreceptor binding have been identified, the details of their interactions and the sequence of events leading to functional recruitment of coreceptor are poorly defined. Recently, HIV-1 variants (JRCSF strain) adapted to use CCR5 lacking the first 18 residues of the N-terminus (referred to as CCR5(Δ18)) have been isolated and characterized [36]. Three mutations in the gp120 V3-loop, S298N, N300Y and T315P, were sufficient to render the virus competent for growing on cells expressing CCR5(Δ18). An additional substitution that resulted in elimination of an N-glycosylation site within the V4-loop further enhanced virus infectivity in cells expressing CCR5(Δ18) [36]. Functional analysis of these JRCSF variants provided evidence that the adaptive mutations lowered the activation energy barrier that restricts gp41 refolding. This allowed gp41 to undergo conformational changes following the low-affinity interaction with the ECL2 region of CCR5(Δ18). Consistent with this interpretation, the adapted viral variants could not employ a double mutant damaged in both its Nt and ECL2 regions [36].

Because HIV-1 infectivity assays do not directly measure Env-mediated fusion, we examined the mechanism of adaptation by directly assessing the Env function in a cell-cell fusion model. We used the adapted JRCSF Env bearing a minimal set of substitutions localized in the V3-loop: S298N, N300Y and T315P (designated NYP). Unlike Env(wt), Env(NYP) was able to induce fusion with cells expressing CCR5(Δ18). Our data confirmed that adaptation to CCR5(Δ18) did not involve significant changes in the binding affinity to CCR5(wt) [36]. Instead, our results suggest that the adaptive mutations cause a more facile triggering of gp41 rather than a compensatory or specific increase in viral affinity for undamaged regions of CCR5(Δ18). In addition, we provide functional evidence that Env(NYP) engages the truncated coreceptor at reduced temperature that does not permit fusion, albeit with much lower efficiency compared to CCR5(wt). We have also shown that the sulfated peptide derived from the Nt of CCR5 rescues the ability of CCR5(Δ18) to support cell fusion and infection mediated by Env(wt). Furthermore, the sulfated peptide allowed Env(wt)-CD4 complexes to engage CCR5(Δ18) upon pre-incubation at sub-threshold temperature. Our data suggest that, after binding the Nt of CCR5, HIV-1 Env acquires the ability to engage other extracellular domains of the coreceptor which, in turn, triggers fusogenic conformational changes in gp41. We propose that the NYP mutations in V3 alter the Env trimers by enabling them to more readily bypass this first step.

## Results

### The adaptation to CCR5(Δ18) does not alter the kinetics of Env-induced fusion or the apparent affinity for wild-type CCR5

Because the Nt of CCR5 is necessary for high affinity binding of HIV-1 gp120(wt) [11,37,30,34,40], it is likely that the adaptive gp120 mutations that enable use of CCR5(Δ18) would compensate by increasing viral interactions with ECL2 or other undamaged regions of the truncated coreceptor. A corollary of this hypothesis is that Env(NYP) would bind CCR5(Δ18) and CCR5(wt) more strongly than Env(wt). An alternative hypothesis, which is more consistent with our previous evidence [36], is that Env(NYP) might be more easily triggered than Env(wt), so that even weak interactions with CCR5(Δ18) would enable gp120(NYP) to release gp41, resulting in a more facile membrane fusion. To test these ideas in the context of membrane fusion, we initially measured the kinetics of fusion of CCR5(wt)-expressing cells with cells containing wild-type or adapted Env. Fusogenic activities of Env(NYP) and wild-type Env (Env(wt)) were compared by expressing them in 293T cells (designated as effector cells) and measuring fusion with target HeLa cells that stably express CD4 at a uniform concentration and comparable surface densities of either wild-type CCR5 or CCR5(Δ18) [36].

**Figure 1:**
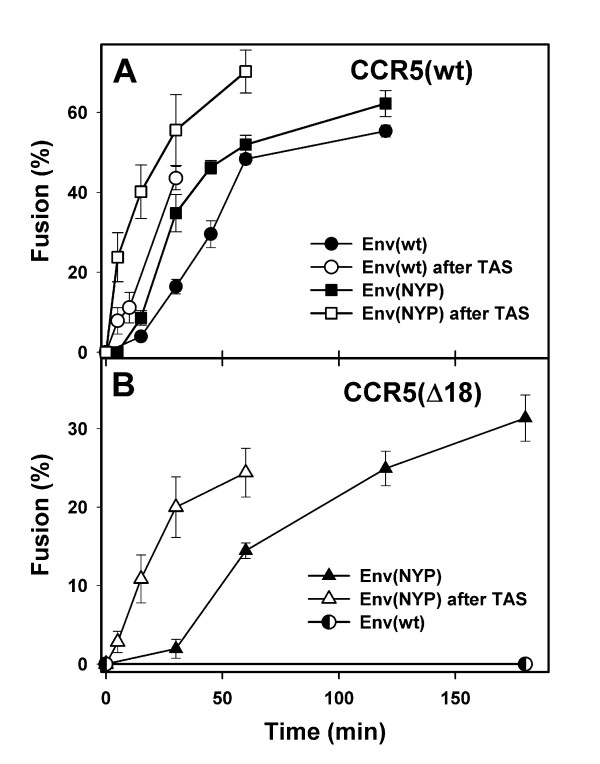
Kinetics of Env(wt)- and Env(NYP)-induced fusion with HeLa-CD4 cells expressing CCR5(wt) (A) or CCR5(Δ18) (B). (A) Fusion with CCR5(wt)-expressing cells induced by Env(wt) and Env(NYP) is shown by filled circles and filled squares, respectively. Fusion after establishing the 18°-TAS (pre-incubation for 2 hr at 18°C) is shown by open circles (Env(wt)) and open squares (Env(NYP)). (B) Fusion with CCR5(Δ18)-expressing cells induced by Env(wt) and by Env(NYP) is shown by semifilled circles and filled triangles, respectively. The kinetics of fusion between Env(NYP)- and CCR5(Δ18)-expressing cells after establishing the 27°-TAS (pre-incubation for 2 hr at 27°C) is shown by open triangles. The experimental points are means ± SE.

Effector and target cells were co-incubated at 37°C and their fusion was stopped at various times by adding a high concentration of fusion inhibitor, C52L [41]. Like other peptides derived from the heptad repeat 2 region of gp41, this recombinant peptide blocks fusion by preventing gp41 refolding into the final 6-helix bundle conformation. The rates of Env(wt)- and Env(NYP)-induced fusion to cells expressing CCR5(wt) and the fraction of cells that fused within 2 hr of co-incubation were similar (Fig. 1A, filled circles and filled squares, respectively). Fusion induced by both Env(wt) and Env(NYP) started after a lag time that is typical for HIV Env-mediated cell-cell fusion [42-46]. We have previously shown that formation of ternary complexes as a result of recruitment of CD4 and coreceptors by HIV Env is primarily responsible for the lag time before fusion [46]. We have found that, when ternary complexes were allowed to form by pre-incubating the effector and target cells at temperatures that were not permissive for fusion, the kinetically advanced temperature-arrested stage (TAS) was created [45-47]. From this intermediate stage, fusion occurred without an appreciable lag time. We examined whether receptor and coreceptor engagement by JRCSF Env(wt) and Env(NYP) is also responsible for the lag time before fusion. Effector and target cells were pre-incubated at sub-threshold temperature (18°C) for 2 hr to establish TAS, and the rate of fusion upon shifting to 37°C was measured. Cell-cell fusion from 18°-TAS progressed without a detectable lag time for both wild-type and adapted Env (Fig. 1A, open circles and open squares). The more synchronous fusion from 18°-TAS indicates that Env(wt) and Env(NYP) had engaged CD4 and CCR5(wt) at sub-threshold temperature [45,46] (see below).

**Figure 2:**
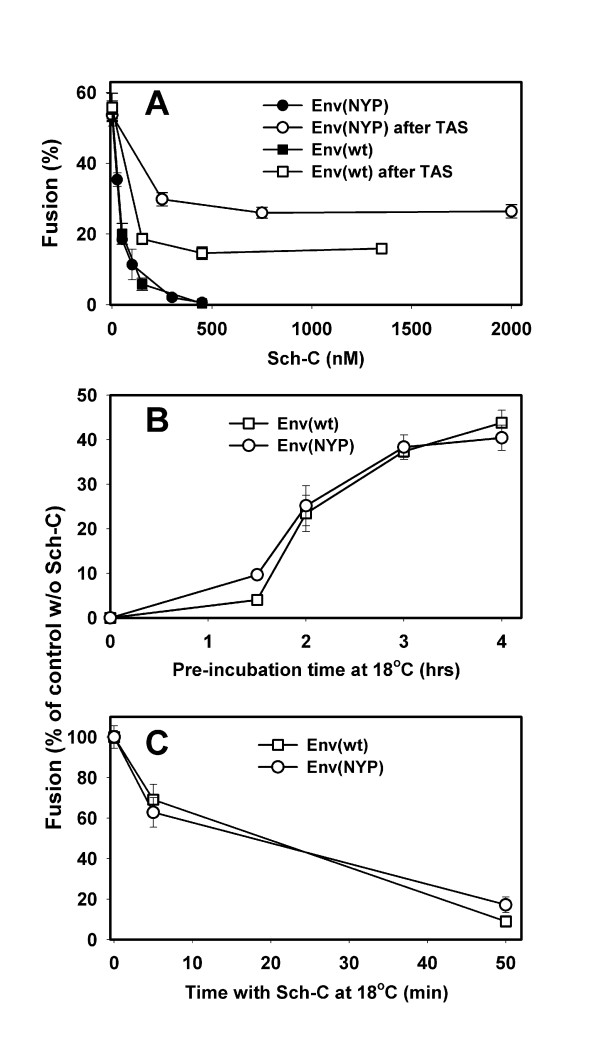
Inhibition of fusion with HeLa-CD4/CCR5(wt)-expressing cells by the CCR5 binding inhibitor, Sch-C. (A) The inhibitory activity of Sch-C was measured upon direct co-culture of effector and target cells at 37°C for 2 hr (filled symbols) and after creating TAS (open symbols). TAS was created by pre-incubating cells at 18°C for 2 hr, and fusion was triggered by additional incubation at 37°C for 1.5 hr. Fusion mediated by Env(wt) and Env(NYP) is shown by squares and circles, respectively. (B) Effector cells expressing Env(wt) (squares) or Env(NYP) (circles) were pre-incubated with HeLa-CD4/CCR5(wt) cells at 18°C for varied times, exposed to 1.35 μM Sch-C for 5 min, and warmed to 37°C for 1.5 hr. (C) Following the creation of TAS (18°C, 2 hr), the cells were incubated for additional 5 or 50 min at 18°C with or without 300 nM Sch-C before raising the temperature to 37°C. The resulting fusogenic activity was normalized to the extent of fusion without the inhibitor. The Env(wt)- and Env(NYP)-induced fusion is shown by squares and circles, respectively.

It has been shown that an apparent binding affinity between Env and CCR5 correlates with the resistance of fusion to coreceptor binding inhibitors [25,27,28]. Accordingly, we used the CCR5 binding inhibitor, Sch-C [48], to evaluate the apparent relative affinity of Env(wt) and Env(NYP) to CCR5(wt). As shown in Fig. 2A, fusion induced by these Envs was equally sensitive to Sch-C (filled circles vs. filled squares). The calculated IC_50 _values for Env(wt) and Env(NYP) were 25 ± 4 nM and 31 ± 4 nM, respectively. Likewise, anti-CCR5 antibodies 2D7 and PA14 nearly identically blocked fluorescent dye redistribution between cells induced by these two Envs (data not shown). These results imply that the adaptation of JRCSF to CCR5(Δ18) did not dramatically alter the apparent affinity to CCR5(wt).

We have previously demonstrated that fusion from TAS was partially resistant to CD4 and coreceptor binding inhibitors [46]. This result implies that ternary Env-CD4-coreceptor complexes had formed during pre-incubation at sub-threshold temperature and that dissociation of these complexes (if any) was slow compared to the rate of fusion upon warming cells to 37°C. When Env(wt)- and Env(NYP)-expressing cells were pre-incubated for 2 hr at 18°C with target cells expressing CD4 and CCR5(wt) (to create TAS), a fraction of cells became resistant to Sch-C. Considerable fusion occurred upon shifting cells to 37°C from TAS in the presence of high doses of the inhibitor added at this stage (Fig. 2A, open symbols). This finding supports the notion that both Envs form complexes with CCR5(wt) at sub-threshold temperature and that a significant fraction of these complexes can fuse in the presence of Sch-C.

Next, we asked whether Env(wt) and Env(NYP) engage CD4 and CCR5(wt) at similar rates, which is likely to reflect the apparent "on-rate" of CCR5 binding. The kinetics of formation of ternary complexes at 18°C was evaluated by measuring the rate of acquisition of resistance to a high concentration of Sch-C added after varied times of pre-incubation. The extents of fusion were quantified after additional incubation at 37°C. Env(wt) and Env(NYP) Env acquired resistance to Sch-C with similar time courses (Fig. 2B). After pre-incubation at reduced temperature for 4 hr, more than 40% of cells expressing either of these Envs exhibited resistance to the drug. Note that at early times (1.5 hr point in Fig. 2B), a slightly greater fraction of Env(NYP)-expressing cells fused with CCR5(wt)-expressing cells in the presence of Sch-C. This observation is consistent with somewhat better protection of Env(NYP)-induced fusion from inhibition by Sch-C added after a 2 hr-incubation at 18°C compared to Env(wt) (Fig. 2A).

In order to assess the stability of the ternary complexes with CD4 and CCR5(wt) formed at sub-threshold temperature, we varied the duration of exposure to Sch-C added at TAS prior to raising the temperature. We reasoned that the time-dependence of inhibition by Sch-C added after CCR5 had been engaged by Env should reflect the "off-rate" for dissociation of coreceptors from Env(wt)-CD4 complexes. Cells captured at 18°-TAS were exposed to 300 nM Sch-C for 5 or 50 min at reduced temperature prior to warming to 37°C. (In control experiments, the incubation steps were identical but Sch-C was omitted). Prolonged pre-incubation with Sch-C at 18°C dramatically reduced the extent of fusion (Fig. 2C). Thus the requisite ternary complexes formed at sub-threshold temperature are reversible. The partial protection against Sch-C at TAS is likely due to the competing processes of dissociation of ternary complexes and somewhat synchronized fusion induced by raising the temperature at this stage (Fig. 1B, open squares). The rates at which fusion diminished with the duration of drug treatment were virtually identical for Env(wt) and Env(NYP), suggesting that the adaptive mutations in the V3-loop did not alter the stability of Env-CD4-CCR5(wt) complexes (Fig. 2C). Collectively, the fusion kinetics data and the indirect measurements of the rates of formation and dissociation of ternary complexes imply that the adaptive mutations in JRCSF Env did not increase its apparent affinity for CCR5(wt).

### Env(NYP) is capable of inducing fusion after low-affinity binding to CCR5(Δ18)

To properly compare the extent and kinetics of fusion supported by wild-type and the N-terminally truncated coreceptor, we utilized HeLa cells expressing comparable levels of CD4 and either CCR5(wt) or CCR5(Δ18) (Figs. 1 and 2). When cells expressing CCR5(Δ18) were used as targets, Env(wt) failed to induce redistribution of cytoplasmic dye (Fig. 1B, semi-filled circles), whereas Env(NYP) was still fusogenic (filled triangles). However, in contrast to CD4^+^/CCR5(wt)^+ ^cells, fusion to CCR5(Δ18)-expressing cells started after a long delay and involved a relatively small fraction of cells. The low efficacy of fusion is in agreement with the similarly reduced infectivity of Env(NYP)-bearing viruses in cells expressing CCR5(Δ18) compared to their infectivity in cells containing wild-type CCR5 [36].

Next, we examined the temperature-dependence of dye transfer with cells expressing CCR5(Δ18). Unlike CCR5-supported fusion that occurred above 18°C [46,47,49], fusion with these cells was not detected at temperatures below 27°C (data not shown). This finding indicates that binding to the truncated coreceptor and/or subsequent refolding of gp41 require an elevated temperature. To distinguish between these possibilities, we tested whether ternary complexes with CCR5(Δ18) can form at 18°C, a temperature that allowed Env(NYP) to engage CD4 and CCR5(wt) (Figs. 1 and 2). Neither the rate of fusion nor the resistance to Sch-C were enhanced significantly after pre-incubation of Env(NYP)- and CCR5(Δ18)-expressing cells for up to 2.5 hr at 18°C (data not shown). On the other hand, pre-incubation at 27°C resulted in formation of a kinetically advanced intermediate, from which fusion proceeded without a detectable lag time (Fig. 1B, open triangles). These results imply that formation of functional ternary complexes between Env(NYP) and CCR5(Δ18) requires higher temperatures than those allowing engagement of CCR5(wt). The requirement for elevated temperature to create TAS indicates that, in order to engage CCR5(Δ18), gp120 must undergo more substantial conformational changes compared those required for binding wild-type CCR5.

**Figure 3:**
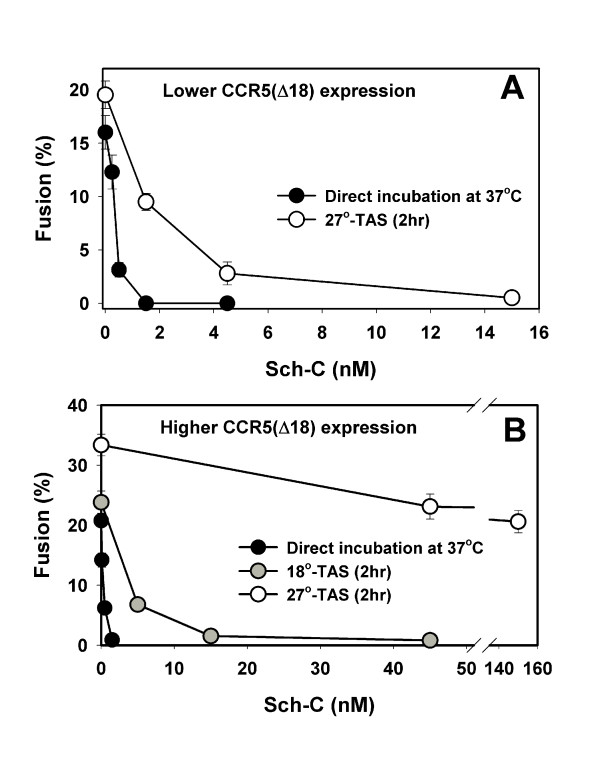
Inhibition of fusion between Env(NYP)- and CD4/CCR5(Δ18)-expressing cells by Sch-C. The extent of fusion upon direct co-incubation at 37°C for 3 hr (filled circles) and after establishing 27°-TAS (open circles) was measured in the presence of different concentrations of Sch-C, as described in the legend to Figure 2. The TAS was created by a 2 hr pre-incubation at 27°C. This temperature was not permissive to fusion with cells expressing a low (R5d18.2, panel A) and relatively high (R5d18.23, panel B) level of the truncated CCR5. Inhibition of fusion by Sch-C added after creating 18°-TAS (2 hr at 18°C) with cells expressing a higher level of CCR5(Δ18) is shown by gray circles.

The elimination of a lag time to fusion upon establishing a 27°-TAS (Fig. 1B) suggests that this lag reflects the time required for formation of ternary Env(NYP)-CD4-CCR5(Δ18) complexes, in agreement with our previous data [46]. The much longer lag time observed for the truncated coreceptor compared to CCR5(wt) is indicative of low-affinity binding between Env(NYP) and CCR5(Δ18). To more directly assess the relative affinity of Env(NYP) to the truncated coreceptor, we measured the sensitivity of fusion to Sch-C. The drug suppressed fusion with cells expressing CCR5(Δ18) with ~150-fold greater efficacy than fusion with CCR5(wt)-expressing cells: the calculated IC_50 _were 0.2 and 31 nM for fusion supported by CCR5(Δ18) and CCR5(wt), respectively (Figs. 3A and 2A, filled circles). Pre-incubation with CCR5(Δ18) cells for 2 hr at 27°C to create TAS rendered fusion induced by Env(NYP) somewhat less sensitive to Sch-C (Fig. 3A, open circles, IC_50 _= 1.1 nM). This result is in contrast with far more marked protection against Sch-C added at 18°-TAS when CCR5(wt)-expressing cells were used as targets (Fig. 2A). Thus, even at somewhat elevated temperature, Env(NYP) does not seem to form stable ternary complexes that would allow Env(NYP) to induce fusion in the presence of Sch-C. On the other hand, the marginal increase in the resistance to Sch-C (Fig. 3) and the accelerated kinetics of fusion (Fig. 1B) from 27°-TAS, indicate that Env(NYP) is capable of low-affinity interactions with CCR5(Δ18) at sub-threshold temperature.

### The Env(NYP)-CCR5(Δ18) binding is highly sensitive to temperature and coreceptor density

The apparent lack of functional recruitment of CCR5(Δ18) by Env(NYP) at 27°C could reflect slow engagement of these coreceptors that requires much longer than 2 hr at this temperature to manifest itself. We therefore tested whether higher levels of CCR5(Δ18) expression would facilitate formation of a drug-resistant intermediate. We employed the R5d18.23 cell line that expressed a 2.4-fold higher density of CCR5(Δ18) compared to R5d18.2 clone [36] used in the above experiments (Fig. 3A). The higher CCR5(Δ18) density did not considerably increase the extent of fusion or the resistance to Sch-C upon direct co-culturing of effector and target cells at 37°C (compare Fig. 3A and 3B, filled circles). But when a 27°-TAS was created with cells expressing a higher density of the truncated coreceptor, a dramatic increase in the resistance to Sch-C was observed. The apparent IC_50 _for Sch-C increased from 0.2 nM upon direct co-incubation of cells at 37°C to 213 nM at TAS (filled vs. open circles). In other words, a 2.4-fold greater density of CCR5(Δ18) increased the resistance to Sch-C added at 27°-TAS more than 200-fold (compare open circles in Fig. 3A and 3B). Note that fusion from 27°-TAS was still fully inhibited by the six-helix bundle-blocking peptide, C52L, added at this stage (data not shown).

The usage of cells expressing a higher density of CCR5(Δ18) permitted us to assess the temperature-dependence of formation of drug-resistant pre-fusion complexes. Figure 3B (gray circles) shows that pre-incubation of cells expressing Env(NYP) with target cells expressing a high amount of CCR5(Δ18) at 18°C resulted in a 9-fold greater resistance to Sch-C (IC_50 _= 1.8 nM) compared to direct fusion at 37°C. Thus protection against Sch-C was far less pronounced at 18°-TAS compared to 27°-TAS: the effective concentration of the drug was 2 orders of magnitude greater at higher pre-incubation temperature. This is in contrast to formation of Sch-C-resistant ternary complexes with wild-type coreceptors that occurs readily within 2 hr at temperature as low as 18°C (Fig. 2A). The remarkably strong dependence of the resistance to Sch-C on CCR5(Δ18) density and on pre-incubation temperature suggests that the Env(NYP)-CCR5(Δ18) binding may be a cooperative process, in agreement with other evidence [50].

Collectively, the accelerated kinetics of fusion and protection against Sch-C after creating TAS show that Env(NYP) does engage CCR5(Δ18) at temperatures that are not permissive to fusion. Moreover, when CCR5(Δ18) was present at sufficiently high density and when the pre-incubation step was carried out at 27°C, Env(NYP) appeared to form ternary complexes with the truncated coreceptor that were capable of progressing to fusion in the presence of high doses of Sch-C. The steep temperature-dependence of CCR5(Δ18) recruitment and the relatively low efficacy of fusion with CCR5(Δ18)-expressing cells (Fig. 1B) suggest that Env(NYP) is less efficient at engaging CCR5(Δ18) compared to CCR5(wt). The above data are consistent with low-affinity interactions between Env(NYP) and CCR5(Δ18) that, under special conditions (27°-TAS), can result in accumulation of active pre-fusion complexes that likely involve multiple cooperatively functioning CCR5(Δ18)s.

### The usage of CCR5(Δ18) increases the apparent residency time of Env(NYP) gp41 in a pre-bundle conformation

**Figure 4:**
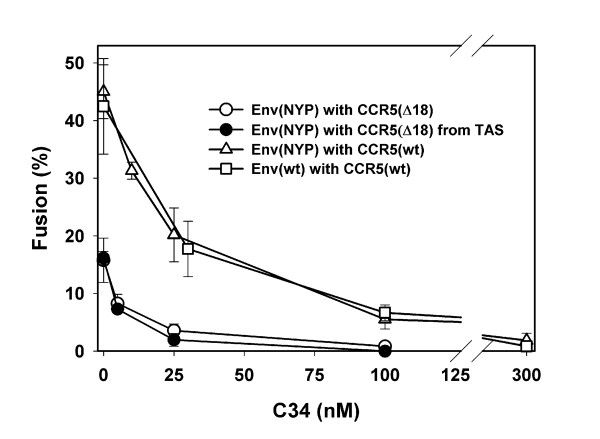
Inhibition of cell-cell fusion by the C34 peptide. The extent of fusion between Env(wt)-expressing (open squares) or Env(NYP)-expressing (open triangles) cells and CD4/CCR5(wt) cells was measured following a 2 hr-incubation at 37°C in presence of indicated concentrations of C34. When CCR5(Δ18)-expressing cells were used as targets, the C34 was added either at the beginning of co-culture with Env(NYP)-expressing cells (2 hr at 37°C, open circles) or after capturing cells at TAS (pre-incubation at 27°C for 2 hr, filled circles). When added at TAS, the C34 was allowed to bind for 5 min before warming the cells to 37°C.

Next, we indirectly evaluated the relative rates of Env(NYP) gp41 refolding into a 6-helix bundle for target cells expressing wild-type CCR5 and CCR5(Δ18). The inhibitory potency of gp41-derived C-peptides (e.g., T-20 or C34) appears to be determined, in part, by the overall time the gp41 spends in a pre-bundle conformation before forming the peptide-resistant 6-helix bundle [27,28]. The C34 peptide blocked Env(wt)- and Env(NYP)-induced fusion to CCR5(wt)-expressing cells equally effectively (Fig. 4), suggesting that Env(NYP) and Env(wt) gp41 proceed through intermediate pre-bundle conformations at similar rates. By contrast, Env(NYP) fusion was 3-fold more sensitive to inhibition by C34 when cells expressing CCR5(Δ18) were used as targets instead of CCR5(wt)-expressing cells (Fig. 4, open circles vs. open triangles). Consistent with our other evidence [36], this finding shows that Env(NYP) can efficiently recognize the CCR5 Nt although it does not require it.

The greater sensitivity to C34 and the overall slower rate of fusion (Fig. 1B) suggest that Env(NYP) gp41 exists in a pre-bundle conformation longer when it is forced to use CCR5(Δ18) instead of CCR5(wt). Because the gp41 coiled coil becomes exposed upon engaging CD4 and coreceptor [51-53,43,45], high sensitivity to C34 is in agreement with low-affinity interactions between Env(NYP) and CCR5(Δ18) that may necessitate recruitment of a greater number of truncated coreceptors in order to initiate conformational changes in gp41. Because the apparent affinity is lower, only a few of the complexes at any moment would have enough CCR5(Δ18)s to refold from a pre-bundle to a 6-helix bundle conformation. Moreover, the weak association might not reduce the activation energy barrier sufficiently to enable the refolding to occur quickly. These effects would slow down the refolding, extending the residency time of gp41 in a pre-bundle conformation compared to fusion supported by wild-type CCR5. We also found that the potency of C34 was not altered after arresting fusion with CCR5(Δ18)-expressing cells at 27°-TAS (Fig. 4, filled circles). This lack of potentiation of C34 activity at TAS indicates that the JRCSF gp41 coiled coil regions are not efficiently exposed upon weak interactions with CCR5(Δ18) at 27°C.

### After binding to CD4, Env(NYP) is more prone to inactivation than Env(wt)

**Figure 5:**
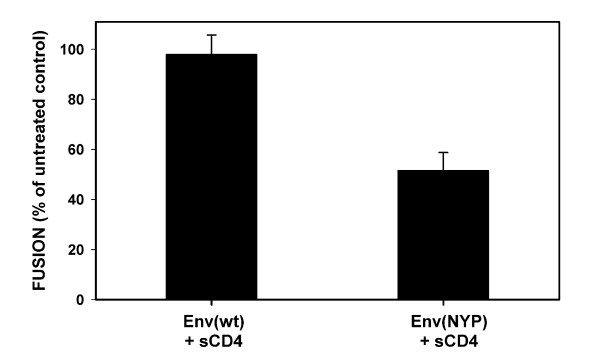
Env(NYP) is more readily inactivated by treatment with sCD4 than Env(wt). Cells expressing either Env(wt) or Env(NYP) were collected from the culture dish using a non-enzymatic cell dissociation solution and treated with 25 μg/ml sCD4 for 30 min at 37°C. Cells were washed twice to remove free sCD4 and co-incubated with HeLa-CD4/CCR5(wt) cells for 2 hr at 37°C. The results are plotted as percentage of fusion observed for untreated effector cells.

The inefficient and/or asynchronous triggering of Env(NYP) upon binding to CCR5(Δ18) (as demonstrated in Fig. 4) may lead to excessive inactivation of Env, consistent with the low extent of fusion observed for CCR5(Δ18)-expressing cells (Figs. 1B and 3). It is, thus, possible that the failure of Env(wt) to use CCR5(Δ18) for fusion is due to a more pronounced inactivation of Env(wt)-CD4 complexes compared to those formed by Env(NYP). However, an alternative interpretation consistent with our other evidence is that the Env(NYP) is less resistant to irreversible conformational change than Env(wt), so that the adapted Env can be more easily triggered by damaged coreceptors such as CCR5(Δ18). In agreement with the latter interpretation, we found that Env(NYP) was much more sensitive to inactivation by soluble CD4 than Env(wt) (Fig 5). Hence, in agreement with the model proposed previously [36], it is likely that the requirements for functional interactions of Env(NYP) with coreceptors that initiate gp41 refolding are less stringent than for Env(wt). In other words, Env(NYP)-CD4 complexes are more prone to undergo irreversible conformational changes than Env(wt)-CD4 complexes (Fig. 5), so that low-affinity interactions with the CCR5 domain(s) other than the Nt are capable of triggering the Env(NYP) gp41 refolding but not refolding of Env(wt).

### Sulfated CCR5 Nt-derived peptides reconstitute the ability of CCR5(Δ18) to support fusion induced by wild-type Env

**Figure 6:**
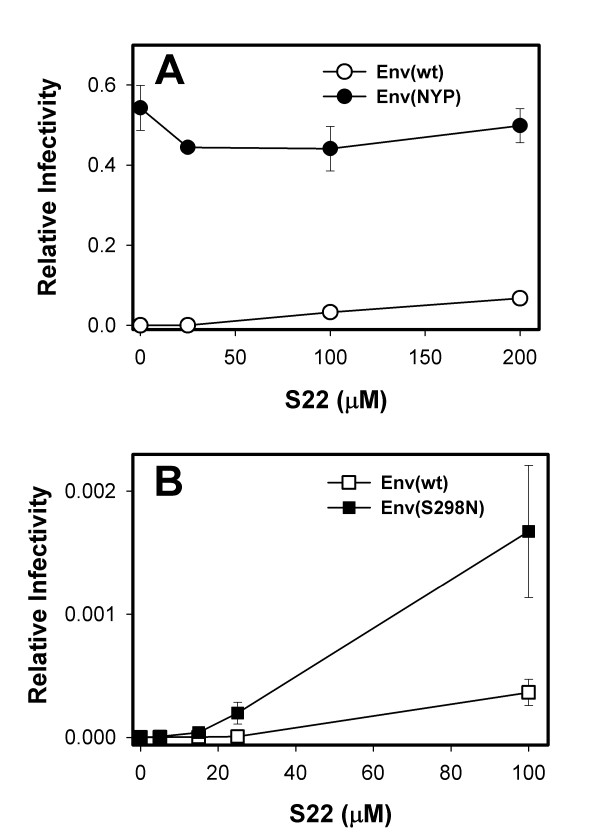
The effect of S22 peptide on virus infectivity. A. Infections of HeLa-CD4 cells expressing CCR5(Δ18) (R5d18.23 cells, 6.6 × 10^4 ^coreceptors/cell) were carried out in the absence and the presence of varying concentrations of the S22 peptide (0, 25, 100, and 200 μM). The replication competent wild-type (open circles) or CCR5(Δ18)-adapted (filled circles) JRCSF isolates were tested. Infections performed in the presence of S22 were normalized to those obtained in cells expressing wild-type CCR5 in the absence of peptide. The graph shows a representative experiment performed in duplicate. Error bars are the range. B. Reconstruction of CCR5(Δ18) function by S22 peptide using a single-cycle infectivity assay. Infectivities of viruses pseudotyped with JRCSF wt (open squares) and S298N mutant (filled squares) were determined in the presence of varied concentrations of the sulfopeptide. Titers were normalized as in panel A. Data points represent averages of two experiments performed in duplicate. Error bars are SE. Note that the overall infectivity of pseudoviruses on these cells was much lower than those obtained for replication-competent viruses (panel B vs. panel A).

Sulfation of tyrosine residues within the Nt of CCR5 is important for CCR5 function as a coreceptor for HIV entry [18]. Sulfated Nt-derived peptides, but not unmodified peptides, were able to inhibit infection by R5-tropic HIV-1 [32,33]. In addition, sulfated peptides rescued the ability of the N-terminally truncated CCR5 to support HIV entry [32,33]. We therefore asked whether the 22 residue-long Nt-derived peptide sulfated at positions 10 and 14 (referred to as S22 [32,33]) will permit the usage of CCR5(Δ18) by Env(wt) for entry and fusion. Replication competent wild-type JRCSF was used to infect HeLa-CD4 cells expressing CCR5(Δ18) in the presence or absence of the S22 peptide. No infections were detected in multiple experiments in the absence of the peptide. In contrast, the sulfopeptide reproducibly caused a small but significant (N = 6; p < 0.05) degree of infectivity by this same virus (Fig 6A, open circles). By comparison, the S22 peptide was unnecessary for infection of these cells by the CCR5(delta 18)-adapted virus and did not have a consistent effect in that case (filled circles).

Even though S22 peptide permitted usage of CCR5(Δ18) by wild-type JRCSF, the efficiency of infection did not exceed 7% of that obtained on cells expressing comparable levels of CCR5(wt) in the absence of the peptide (Fig. 6A). In contrast, ADA and YU2 strains of HIV-1 have been reported to efficiently utilize S22 peptide to infect cells expressing N-terminally truncated CCR5 [23]. The determinants for binding of the S22 peptide to monomeric gp120 have been previously mapped using the HIV-1 JRFL isolate [23]. These studies implicated the relatively conserved stem region of the V3-loop in binding the sulfated peptide. Comparison of the sequences of JRCSF and JRFL isolates revealed that their V3-loops were identical except that JRCSF had serine at position 298 of the V3 loop stem instead of the consensus asparagine residue found in JRFL, ADA, YU2 and other Clade B viruses.

Note that the S298N substitution is one of the three adaptive mutations in JRCSF Env permitting the usage of CCR5(Y14N) and CCR5(Δ18) for entry [36]. This finding prompted us to assess the ability of JRCSF S298N mutant to use the S22 peptide and CCR5(Δ18) "in-trans" for virus entry. We have made pseudoviruses bearing wild-type or S298N JRCSF Env and compared their infectivities in the presence of the sulfopeptide. Figure 6B shows that the S298N mutation alone in the context of the JRCSF gp120 enhanced the S22-dependent infection of cells expressing CCR5(Δ18). Thus, poor utilization of the S22 peptide by JRCSF compared to other clade B viruses is, at least in part, due to the presence of serine 298 in its V3-loop.

**Figure 7:**
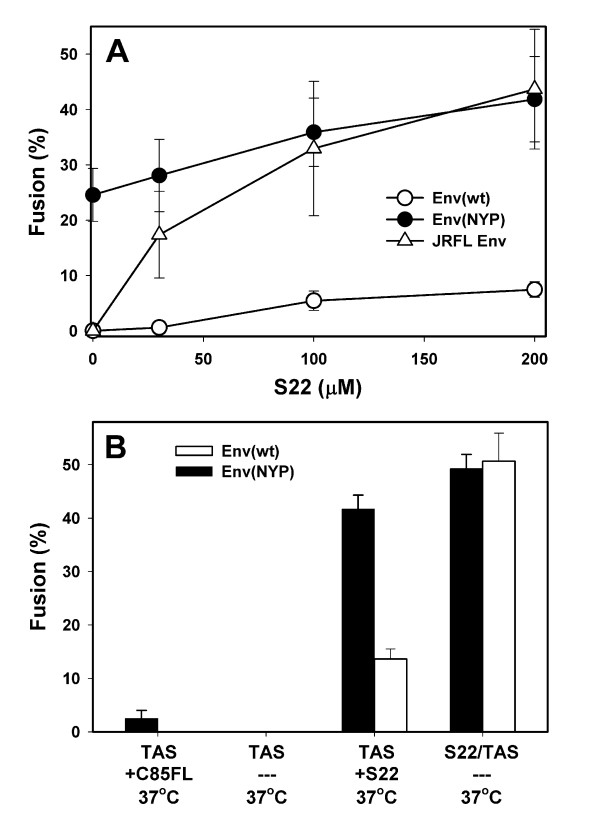
Rescue of the CCR5(Δ18) coreceptor function by the S22 peptide. (A) Cells expressing Env(wt) (open circles) or Env(NYP) (filled circles) were co-cultured with HeLa-CD4 cells expressing a relatively high density of CCR5(Δ18) (the R5d18.23 line) for 3 hr at 37°C in the presence or in absence of S22. For comparison, cell-cell fusion induced by JRFL Env in the presence of varied concentrations of the S22 peptide is shown by open triangles. (B) Fusion between Env(NYP)- (filled bars) or Env(wt)-expressing (open bars) cells and CD4/CCR5(Δ18) cells after establishing a 27°-TAS. Two μM of C52L was added (first column) or not added (second column) after creating TAS (27°C, 2 hr) prior to warming cells to 37°C and incubating for additional 2 hr. Third column shows fusion observed when 200 μM S22 was present during the last 30 min of a 2 hr pre-incubation at 27°C required to create TAS. Alternatively, a 27°-TAS was created in the presence of 200 μM S22 peptide, and cells were additionally incubated for 2 hr at 37°C (fourth column).

The S22 peptide also rescued the CCR5(Δ18) function in a cell-cell fusion assay, as evidenced by considerable redistribution of cytoplasmic dye between Env(wt)- and CCR5(Δ18)-expressing cells (Fig. 7A, open circles). The sulfopeptide also augmented fusion induced by Env(NYP) (filled circles), showing that interaction with both S22 and CCR5(Δ18) is beneficial, but not absolutely necessary for Env(NYP) fusion activity. Note, however, that even at S22 concentrations as high as 200 μM, the extent of fusion of Env(wt) was still lower than that induced by Env(NYP) in the absence of the sulfopeptide. Overall, the S22-dependent Env(wt) fusion with CCR5(Δ18)-expressing cells reached only 15% of the level supported by CCR5(wt)-expressing cells (compare Fig. 1A, filled circles, to Fig. 7A, open circles). Thus, wild-type JRCSF Env inefficiently utilizes the S22 and CCR5(Δ18) "in-trans" to initiate infection (Fig. 6) or to induce cell-cell fusion.

Based on our infectivity data, JRFL Env that has asparagine at position 298 is expected to utilize S22 more efficiently for infecting cells expressing CCR5(Δ18). We, therefore, assessed the ability of S22 peptide to reconstitute JRFL Env-induced fusion with cells expressing the truncated coreceptor. Whereas cell-cell fusion was not detected in the absence of S22, co-incubation of JRFL- and CCR5(Δ18)-expressing cells in the presence of varied concentrations of S22 led to efficient dye redistribution (Fig. 7A). The extent of JRFL Env-induced fusion with CCR5(Δ18) cells in the presence of 200 μM of S22 reached 50–60% of fusion supported by comparable levels of wild-type CCR5 (data not shown). The more efficient utilization of the S22 by JRFL compared to JRCSF provides further support to our conclusion that the S298N substitution in the V3 loop may improve binding of the sulfopeptide.

### Pre-incubation with the S22 peptide at sub-threshold temperature enhances the ability of Env(wt) to use CCR5(Δ18)

We reasoned that if weak binding of S22 to gp120-CD4 reduces the probability of engaging the truncated coreceptor, the efficacy of fusion should be improved by extending the window of opportunity for coreceptor binding. To test this notion, we pre-incubated Env(wt)- and CCR5(Δ18)-expressing cells at sub-threshold temperature (27°C, 2 hr to create TAS) in the presence or in absence of the S22, followed by additional incubation at 37°C to induce fusion. Cell-cell fusion was not evident when S22 was not present in the incubation medium (Fig. 7B, second column). Addition of S22 (200 μM) during the last 30 min of pre-incubation at 27°C resulted in significant cell-cell fusion, but the extent of dye redistribution was comparable to that obtained upon direct incubation at 37°C using the same concentration of S22 (Fig. 7A vs. 7B, third column). In sharp contrast, when the S22 was present throughout the pre-incubation step (2 hr, 27°C), fusion was markedly enhanced. As much as 50% of cells transfected with Env(wt) fused with cells expressing CCR5(Δ18) (Fig. 7B, fourth column), approaching the extent of dye redistribution supported by CCR5(wt) (Fig. 1A). This finding implies that Env(wt) can recruit CD4, S22 and the damaged coreceptor at sub-threshold temperature. Parallel measurements of Env(NYP)-induced fusion with CCR5(Δ18)-expressing cells revealed that S22 only modestly enhanced cytoplasmic dye redistribution, irrespective of the experimental protocol (Fig. 7B, filled bars). To summarize, wild-type JRCSF Env becomes competent to induce fusion when sufficient time is provided for binding S22 and CCR5(Δ18) at sub-threshold temperature.

Even if S22 binds to JRCSF Env with low affinity, this process is unlikely to be rate-limiting for fusion or for formation of fusion-competent complexes at 27°C, because these peptides bind monomeric gp120-CD4 complexes very quickly [23]. We therefore propose that, the Env-CD4-S22 complexes form relatively quickly at reduced temperature while subsequent engagement of CCR5(Δ18) occurs slowly, taking hours to reach completion. This would explain why the stimulating effect of S22 is marginal after a shorter pre-incubation at 27°C (Fig. 7B, third column). We assume that Env does not readily inactivate at sub-threshold temperature, permitting the S22 binding and slow formation of functional ternary complexes. In contrast, at 37°C (even in the presence of S22), JRCSF Env appears to lose activity before it forms ternary complexes and becomes fusion-competent.

## Discussion

Our results indicate that adaptation of HIV-1 Env to use CCR5(Δ18) for entry does not involve a tighter interaction with the wild-type CCR5. The unaltered affinity to CCR5(wt) is supported by similarities in the following characteristics of fusion mediated by Env(wt) and Env(NYP) glycoproteins: (i) the rate and extent of cytoplasmic dye redistribution; (ii) the resistance to CCR5 binding inhibitors; (iii) the ability to engage CCR5 at reduced temperature; and (iv) the apparent stability of ternary Env-CD4-CCR5 complexes in the presence of Sch-C. The finding that fusion from 18°-TAS was virtually abrogated after prolonged exposure to Sch-C showed that Env reversibly engaged CCR5 at reduced temperature (Fig. 2C). Under our conditions, half of the pre-formed complexes dissociated (i.e., were not able to proceed to fusion) within 25 min in the presence of Sch-C.

In contrast to fusion supported by CCR5(wt), Env(NYP)-induced fusion with CCR5(Δ18)-expressing cells appears to occur through low-affinity interactions with the truncated coreceptor. First, fusion supported by CCR5(Δ18) is extremely sensitive to inhibition by Sch-C. Second, the unusually long lag time before fusion (Fig. 1B) is consistent with the reduced affinity to the truncated coreceptor (low apparent "on-rate") that would slow down the formation of ternary complexes. Supporting the notion of low-affinity interactions with CCR5(Δ18) is the demonstration that CCR5(Δ4) construct supported JRFL infection but did not permit detectable gp120 binding (apparent binding affinity >21 nM) [54].

Even for the adapted Env, the temperature that permitted CCR5(Δ18) engagement was considerably higher than that required for engaging CCR5(wt) (27° vs. 18°C). This result suggests that gp120 must undergo additional temperature-dependent conformational changes in order to form a functional pre-fusion complex with the truncated coreceptor. We have previously obtained evidence that formation of ternary complexes with CD4 and CXCR4 is highly temperature-dependent with the temperature coefficient, Q_10_, around 10 [46]. In this work, we observed a much steeper temperature-dependence (Q_10_~100) for recruitment of CCR5(Δ18) by JRCSF-derived Env(NYP). The slow and highly temperature-dependent engagement of coreceptor during cell-cell fusion is in contrast with binding of monomeric JRFL gp120 to CCR5 that occurred quickly and exhibited weak dependence on temperature [55].

The remarkable temperature-sensitivity of ternary complex formation (Fig. 3B) may be indicative of cooperative conformational changes in Env involved in formation of pre-fusion complexes with the truncated coreceptor. In addition, the extremely steep dependence of formation of Env(NYP)-CD4-CCR5(Δ18) complexes on coreceptor density suggests that the requisite number of truncated coreceptors in functional pre-fusion complexes may be greater than the number of wild-type CCR5. We cannot rule out the possibility that markedly different temperature- and coreceptor density- requirements for TAS formation by Env(wt) and Env(NYP) are, at least in part, due to differences in the propensity of ternary complexes to form higher order oligomeric assemblies. However, we do not anticipate that deletion of the CCR5 Nt would alter the ability of Env (or of ternary complexes) to oligomerize. On the other hand, if a greater number of CCR5(Δ18) is required to form pre-fusion complexes, this process may be more critically dependent on the lateral mobility of truncated coreceptors.

The finding that inactivation of Env(NYP)-sCD4 complexes is more prominent compared to Env(wt) (Fig. 4) supports the view that adapted Env is in the "hair-trigger" state, ready to undergo conformational changes upon low-affinity interactions with truncated coreceptor [36]. Thus a few adaptive mutations localized within the V3-loop appear to alter the overall stability of Env bound to CD4, consistent with the fact that the V3-loop is the major determinant of the ability of sCD4 to neutralize HIV [56]. Taken together, these results imply that local changes in the V3-loop can modulate the global stability of Env-CD4 complexes. We speculate that the adaptive mutations diminish the activation energy barrier for Env(NYP) gp41 refolding to the point where it can be triggered by low-affinity interactions with the ECL2 alone.

There are many precedents for the usage of low-affinity coreceptors for HIV entry: the laboratory adapted X4-tropic HIV-1 Envs bind to CXCR4 with low affinity but are capable of promoting efficient fusion. The apparent binding constant for monomeric X4 gp120 (200–500 nM [57,58]) is ~100-fold lower than that for R5 gp120 (4–15 nM [55,59]). It is thus likely that laboratory adapted X4-tropic Env have evolved to use low-affinity coreceptors through lowering the activation barrier for gp41 refolding. Supporting this idea is the fact that, overall, the X4-tropic isolates are less reliant on the Nt region of coreceptor than R5 isolates [32,35].

Recent studies suggest that Env-coreceptor interaction occurs in multiple steps [8,14,16]. It is thought that the stem of the V3-loop and the bridging sheet of gp120 bind to the Nt of CCR5, whereas the tip of the protruding V3-loop interacts with other determinants of CCR5, most likely ECL2 [8,16,24]. The observation that anti-ECL2 antibodies potently block infection, while moderately affecting the binding of monomeric gp120 to CCR5 [11,37,38], argues that high-affinity binding of gp120 involves the Nt domain, while interactions with the ECL2 trigger fusion. Indeed, whereas binding of monomeric gp120 to CCR5(wt)-expressing cells in the presence of sCD4 could be readily observed, binding to CCR5(Δ18)-expressing cells was not evident, even in the presence of S22 peptide in the medium [32]. Moreover, substitution of critical tyrosines within the CCR5 Nt reduces the binding of gp120-CD4 complexes to mutant coreceptors [30]. In addition, CXCR4, CCR1 and CCR2b chimeras bearing the N-terminal domain of CCR5 have been shown to support entry of R5-tropic viruses [19,60]. These results indicate that the specificity of R5 gp120 interactions with coreceptors is determined, at least in part, by the Nt region.

The soluble S22 peptide reconstitutes the ability of the N-terminally truncated CCR5 to support HIV-1 entry [32] and cell-cell fusion (this work). However, this peptide only partially restored the ability of CCR5(Δ18) to function as a coreceptor for wild-type JRCSF Env (Figs. 6 and 7); the resultant fusion was far less efficient than that supported by CCR5(wt). Unexpectedly, pre-incubation with the S22 peptide at sub-threshold temperature greatly enhanced the efficacy of Env(wt)-induced fusion with CCR5(Δ18) cells (Fig. 7B). Creation of TAS also improved the efficacy of Env(NYP)-mediated fusion with CCR5(Δ18)-expressing cells, albeit to a lesser extent (Figs. 3 and 7B). The improved efficacy of Env(wt) fusion in these experiments is likely due to stabilization of Env-CD4 and/or Env-CD4-S22-CCR5(Δ18) complexes at sub-threshold temperature that reduces the probability of Env inactivation. These data indicate that S22 binding is a prerequisite for wild-type gp120-CCR5(Δ18) interactions. The surface plasmon resonance data [8,33] showed that the "on" and "off" rates of S22 binding to monomeric gp120 were very fast. Thus the S22 binding step should not be limiting for formation of functional pre-fusion complexes at reduced temperature. The slow rate of formation of ternary complexes (based on resistance to Sch-C) is most likely determined by subsequent engagement of CCR5(Δ18) by the CD4- and S22-primed gp120. We propose that this sequence of events occurs upon binding to wild-type CCR5 – first gp120 engages the Nt and then interacts with ECL2. The latter step triggers conformational changes in gp41. It appears that the Nt region interacts with the bridging sheet and the base of the V3-loop, whereas the ECL2 domain binds to the tip of V3-loop [8,16,23]. This model is supported by the molecular dynamic simulation of gp120-coreceptor interactions [24] and by the crystal structure of gp120 with the intact V3-loop [16].

## Conclusion

Our data imply that adaptation of JRCSF Env to CCR5(Δ18) does not occur through increasing the affinity to CCR5 determinants other than the Nt. Rather, the adaptation results in lowering the activation energy barrier for gp41 refolding which permits the usage of damaged, low affinity coreceptor. We also found that wild-type Env can slowly associate with CD4 and CCR5(Δ18) at sub-threshold temperature in the presence, but not in the absence of the sulfated Nt-derived peptide. This finding suggests a similar sequence of events for fusion induced by wild-type HIV-1 Env with cells expressing CCR5(wt): engaging the Nt of CCR5 permits gp120-ECL2 interaction, which, in turn, releases the gp120 hold on gp41.

## Methods

### Cell lines and transient expression of HIV-1 Env

HeLa cell derivatives expressing CD4 and either CCR5 or CCR5(Δ18) (JC.6, R5d18.2 and R5d18.23) were maintained in DMEM supplemented with 10% FBS (Hyclone, Logan, UT), penicillin and streptomycin, as described in [36]. In addition to the above supplements, the growth medium for HEK 293T cells contained 0.5 mg/ml G418 (Sigma). The R5d18.2 and R5d18.23 lines express on average 1.5 · 10^5 ^molecules of CD4 and 2.7 · 10^4 ^or 6.6 · 10^4 ^molecules of CCR5(Δ18) per cell, respectively [36]. The JC.6 cells express CD4 and wild-type CCR5 at levels similar to those expressed in R5d18.2 cells [61]. To express HIV-1 JRCSF Env, a 6 cm culture dish of HEK 293T cells was transfected with 5 μg of pcDNA3.0 rev/JRCSF or pcDNA3.0rev/NYP plasmid [62] and of 2.5 μg of cRev plasmid. These plasmids encode JRCSF Env and its NYP variant with S298N, N300Y and T315P mutations in the V3-loop [36]. Cells were transfected using the calcium phosphate precipitation method and were used for fusion experiments 42–45 hr post transfection. Both Env(wt) and Env(NYP) proteins were expressed in 293T cells at comparable levels, as determined by flow cytometry (data not shown).

### Reagents

Small molecule CCR5 binding inhibitors, Sch-C and AD101, were kindly provided by Dr. Julie Strizki (Schering-Plough, Kenilworth, NJ). The gp41-derived C34 peptide (residues 628–661) was synthesized by Macromolecular Resources (Fort Collins, CO), and a 55 residue-long recombinant peptide, C52L, was a generous gift from Dr. Min Lu (Cornell University). The C52L peptide (NHTTWMEWDREINNYTSLIHSLIEESQ NLQEKNEQELLELDKWASLWNWFNIKIK) that encompasses virtually the entire second heptad repeat domain of gp41 [41] has been routinely used to stop the fusion reaction at desired time points. The soluble CD4 (sCD4) was obtained from Progenics Pharmaceutical (Tarrytown, NY). Fluorescent dyes, calcein AM and CellTracker™ Blue (CMAC, 7-amino-4-chloromethylcoumarin) were purchased from Molecular Probes/Invitrogen (Carlsbad, CA). A 22-residue peptide derived from the N-terminal domain of human CCR5, S22 [32] containing sulfated tyrosines at positions 10 and 14 was synthesized by American Peptide Company (Sunnyvale, CA) and was 98% pure, as judged by HPLC.

### Virus infection

Infections using wild-type replication competent HIV-1 JRCSF or variants adapted to use Δ18R5 were performed as described [61]. Target cells were HeLa-CD4 cells expressing wild-type CCR5 or a cell clone expressing Δ18R5 (6.6 · 10^4 ^molecules/cell). Infections were performed in the absence or in the presence of varying concentrations of S22. Infectious titers were obtained by the focal infectivity method of Chesebro [63,64]. HIV-*gpt *pseudotyped viruses bearing wild-type or mutant envelopes were produced and titered as described in [36].

### Cell-cell fusion

Fluorescence microscopy-based cell fusion assay has been described in detail elsewhere [45]. Briefly, effector 293T cells expressing HIV-1 Env, were labeled with calcein AM (green emission) and co-incubated with target cells loaded with CMAC (blue emission) to allow cell fusion. Target cells expressed CD4 and either CCR5 (JC.6 line) or CCR5(Δ18) (R5d18.2 and R5d18.23 lines). Cells were co-incubated in HEPES-buffered DMEM supplemented with 2 mg/ml BSA. A minimum of 100 cell pairs were inspected for every measurement and the extent of fusion under various experimental conditions was determined from at least 2 independent experiments containing duplicate measurements. Fusion was quantified in several image fields by counting the blue-green cells (containing both calcein and CMAC) and the number of unfused blue cells that were in contact with at least one fusion partner, using standard fluorescein and DAPI filters on the Zeiss Axiovert 200 microscope (Carl Zeiss, Thornwood, NY). The extent of fusion was plotted as per cent of fused cells out of the sum number of fused cells and unfused target cells that were in contact with at least one effector cell [45]. The efficacy of fusion inhibitors (IC_50_) was determined as described in [47] by fitting the experimental data to one site competition model.

The kinetics of cell fusion was measured by stopping the process after varied times of cell co-incubation at 37°C. This was achieved by adding 2 μM of C52L, a concentration that exceeded the fully inhibitory dose by ~3-fold, to the cell incubation medium. In order to capture fusion at a temperature-arrested stage (TAS), the effector and target cells were pre-incubated for 2–2.5 hr at either 18°C (for target cells expressing CCR5(wt)) or 27°C (for CCR5(Δ18)-expressing cells), unless stated otherwise. From TAS, fusion was induced by additional incubation for 1.5–2 hr at 37°C. The acquisition of resistance to Sch-C as a function of pre-incubation time at reduced temperature was measured by adding 1.35 μM Sch-C at indicated times of cell co-incubation. Unless stated otherwise, cells were exposed to high doses of Sch-C for 5 min at non-permissive temperature prior to triggering fusion by warming cells to 37°C.

## Abbreviations

C52L, recombinant peptide derived from the second heptad repeat domain of HIV-1 gp41; CCR5(Δ18), N-terminally truncated CCR5; ECL2, extracellular loop 2 of a chemokine receptor; Nt, amino-terminal segment of a chemokine receptor; Env, envelope glycoprotein; NYP, mutant adapted to grow on N-terminally truncated CCR5; S22, sulfated 22-residue-long peptide derived from the N-terminus of CCR5; TAS, temperature-arrested stage of HIV-1 Env-induced fusion; wt, wild-type.

## Competing interests

The author(s) declare that they have no competing interests.

## Authors' contributions

GM conceived this work, designed the experiments, analyzed the cell-cell fusion data and drafted the manuscript. EP carried out the infectivity experiments and helped to draft the manuscript. DK participated in the design of experiments, interpreting the results and writing the manuscript. All authors read and approved the final manuscript.
